# ASO Author Reflections: Onward with Sentinel Lymph Node Omission: Implications of SOUND and INSEMA Trials for Mastectomy Patients

**DOI:** 10.1245/s10434-026-19744-5

**Published:** 2026-05-12

**Authors:** Jennifer H. Chen, Taiwo Adesoye

**Affiliations:** Surgical Oncology, University of Texas MD Anderson Cancer Center, Houston, USA

## Past

The de-escalation of axillary surgery in early breast cancer has continued to evolve toward more selective, and risk-adapted approaches without compromising oncologic safety. Sentinel lymph node biopsy (SLNB) remains the standard of care for axillary staging in patients with clinically node-negative (cN0) disease. However, results from INSEMA and SOUND trials demonstrated that omission of SLNB was noninferior to SLNB among select patients with small (≤2 cm), cN0 tumors, a negative axillary ultrasound, and receiving breast conservation therapy with whole breast irradiation (WBI).^1,2^ This approach has largely been limited to patients undergoing breast conservation therapy due to the presumed therapeutic benefit of incidental axillary radiation delivered during WBI. We examined whether findings from INSEMA and SOUND may be extended to patients undergoing mastectomy.

## Present

This study evaluated the SLNB de-escalation framework among patients with clinical T1N0M0, HR+/HER2− disease undergoing either breast conservation therapy or mastectomy.^3^ Importantly, all patients were confirmed to be clinically node-negative with a baseline axillary ultrasound. Our results demonstrated that rates of sentinel lymph node positivity remained low at 7.7% regardless of the extent of surgical resection performed and rates of pN2 disease were low (<0.5%). These findings suggest that de-escalation of axillary surgery may be appropriate for select HR+/HER2− patients undergoing mastectomy in the setting of a negative axillary ultrasound. However, axillary staging among patients undergoing mastectomy influenced decisions regarding adjuvant radiation therapy and chemotherapy. These findings underscore the importance of multidisciplinary coordination in the era of treatment de-escalation, as the de-escalation of one treatment inevitably impacts decision making for other therapies. Although omission of SLNB may be oncologically safe in select patients, the absence of nodal information may limit eligibility for certain therapies and clinical trials that remain restricted to node-negative populations.^4,5^ Furthermore, absence of axillary staging may restrict patients from therapies such as CDK4/6 inhibitors, which were approved after completion of SOUND and INSEMA trials.^6,7^ Importantly, INSEMA radiotherapy quality assurance data demonstrated that regional nodal irradiation was rarely used (<1%) in the no SLNB arm and incidental axillary radiation was variable and mostly limited to level 1.^8^ Despite this, oncologic outcomes remained noninferior, including among patients who did not receive postoperative radiation. These findings suggest that incidental radiation may not be the primary driver of oncologic safety in SLNB omission.

## Future

Findings from this study establish the basis for future prospective evaluation of axillary surgery de-escalation in patient with early-stage HR+/HER2− breast cancer undergoing mastectomy. As treatment decisions increasingly reflect tumor biology, reliance on nodal status may diminish. Studies such as IDEA trial demonstrate that genomically selected patients can achieve excellent outcomes with omission of radiation while TAILOR-RT is evaluating omission of regional nodal irradiation even in node-positive disease, further challenging the need for axillary surgery to guide adjuvant therapy.^5,9^ Future efforts should focus on developing risk stratification tools to guide omission of axillary surgery, alongside coordinated de-escalation across surgical, radiation, and systemic therapies. As breast cancer care becomes increasingly personalized, shared decision-making based on tumor biology and patient preferences will be critical to optimize quality of life without compromising oncologic outcomes.
